# The Advantages of a Tapered Whisker

**DOI:** 10.1371/journal.pone.0008806

**Published:** 2010-01-20

**Authors:** Christopher M. Williams, Eric M. Kramer

**Affiliations:** Physics Department, Bard College at Simon's Rock, Great Barrington, Massachusetts, United States of America; University of East Piedmont, Italy

## Abstract

The role of facial vibrissae (whiskers) in the behavior of terrestrial mammals is principally as a supplement or substitute for short-distance vision. Each whisker in the array functions as a mechanical transducer, conveying forces applied along the shaft to mechanoreceptors in the follicle at the whisker base. Subsequent processing of mechanoreceptor output in the trigeminal nucleus and somatosensory cortex allows high accuracy discriminations of object distance, direction, and surface texture. The whiskers of terrestrial mammals are tapered and approximately circular in cross section. We characterize the taper of whiskers in nine mammal species, measure the mechanical deflection of isolated felid whiskers, and discuss the mechanics of a single whisker under static and oscillatory deflections. We argue that a tapered whisker provides some advantages for tactile perception (as compared to a hypothetical untapered whisker), and that this may explain why the taper has been preserved during the evolution of terrestrial mammals.

## Introduction

Mammalian vibrissae (whiskers) are sophisticated sense organs, used as a supplement for short-range vision, and in some cases to detect vibrations in air or water [1], [2], [3]. Vibrissae are easily distinguished from other kinds of body hair by their large basal diameter and by the presence of a heavily innervated and vascularized follicle (called the follicle-sinus complex, or FSC). Contact between the whisker and an object produces activity in hundreds of mechanoreceptors in the FSC. The angle at which each whisker projects from the face is under voluntary control in many species, due to the attachment of striated muscle to the FSC. Rats, in particular, probe their environment with a stereotypical “whisking” movement – sweeping the whiskers forward and back with a period ∼0.1 s [4], [5]. However, most species simply protract or retract the whiskers as needed (e.g. seals, cats), and some species have whiskers that are immobile (e.g., horses, cows) [3], [6].

In recent years, the large facial vibrissae of the rat have become a model system in sensory neuroscience. Experiments have shown that rats can perform remarkable feats of tactile discrimination in comparison tasks involving small differences in object distance, direction, or surface texture. Using just their vibrissae, rats can distinguish between aperture openings of 62 and 65 mm diameter [7], between the placement of vertical posts that differ by as little as ∼1° in direction to the snout [8], between a smooth surface and one with grooves just 30 µm deep [9], and between sandpapers of different coarseness [10]. Discriminations with high accuracy typically require the integration of information from multiple whiskers, and performance decreases if some whiskers are cut [4], [7].

In parallel with behavioral research, the neural correlates of various whisker stimuli have been observed. The mechanoreceptors of the FSC send signals to the primary somatosensory cortex (S1), via the trigeminal ganglion (Vg) and the thalamus. In the S1, each whisker is represented by a distinct column of neurons called a “barrel”. Measurements of neuron spike patterns in the Vg and in S1 barrels have revealed populations of cells that encode the kinematic state of the whisker (protraction or retraction, etc) [11], [12], contact between the whisker and an object [12], the direction of whisker deflection during contact [13], the distance of an object from the face during contact [14], vibrations of the whisker in certain frequency ranges [15], [16], stick-slip frictional contact [17], and surface roughness [18], [19]. Thus, the wide range of discrimination tasks performed by rats is reflected by an equally wide diversity of neural population and spike encoding schemes.

The whisker functions as a mechanical transducer, converting forces applied along the shaft to tissue forces in the FSC, where the mechanoreceptors are located [20]. The specific mechanics of the whisker as a transducer are still unclear, but there are several published models. First and most simply, the whisker may function as an approximately rigid beam. Contact in this case would be read as an “all-or-nothing” signal, and the direction of whisker emergence from the FSC would indicate the direction of the object (possibly after a correction for whisker curvature) [12], [21]. Brecht et al. [21] published an early model for how this might work in practice. If a whisker of length L_1_ is in contact with an object and a shorter adjacent whisker of length L_2_ is not, then the object is presumably at a distance between L_1_ and L_2_. Subsequent work has revealed this to be a simplification, but the basic view of the whisker array as a sensor for object distance and direction remains sound [7], [8].

A revised view of whisker mechanics, in its role as a transducer, was provided by Kaneko [22], who noted that a flexible whisker has advantages over a rigid whisker for distance discrimination tasks. Namely, the whisker can remain in contact with an object while the base of the whisker is protracted through a range of angles by the muscles of the follicle. For a given net protraction angle, objects closer to the whisker base will generate larger mechanical stresses in the tissues of the FSC. The ratio of the stress to the amount of protraction – called variously the “rotational stiffness” or “rotational compliance” – can be directly related to the distance of object contact. In this way, Kaneko [22] and subsequently Solomon et al. [23] suggested that a single flexible whisker can be used to extract information about contact distance. Thus, an array of flexible whiskers can in principle perform better than an equivalent array of rigid whiskers. This proposal remains speculative, due to a lack of detailed data on stresses in the FSC. However, the rotational stiffness hypothesis would seem to be the most direct explanation for the observation of Szwed et al. [14], who found that some Vg neurons encode the contact distance along isolated whiskers.

The role of the whisker as a mechanical oscillator is also relevant for tactile perception [16], [24], [25], [26]. Both Hartmann et al. [24] and Neimark et al. [25] measured the response of isolated whiskers to mechanical vibrations. Both found that whiskers exhibit resonant phenomena, which is to say that the whisker itself vibrated most strongly in response to a narrow range of driving frequencies. Neimark et al. [25], then proposed that this resonance is a likely mechanism for texture discrimination. Namely, a whisker tip dragged over a rough surface will only vibrate detectably if the rate at which surface features deflect the tip matches a resonant frequency of the whisker. An array of whiskers with a range of resonant frequencies, whisked over an object, might convert texture information into components with different frequencies, analogous to the way the ear analyzes sound. In agreement with the hypothesized role of the whisker as a resonator, Andermann et al. [15] identified populations of neurons in the Vg and S1 that respond to whisker vibrations only within a narrow band of frequencies (+/−40 Hz) centered at resonance.

In this paper we are interested in the possible importance of whisker taper for its role as a transducer. There are widespread reports of whisker taper in the literature, but few quantitative studies. Ahl [27] made a microscopic survey of vibrissae in 46 species of Sciuridae (squirrels) and reported that “the tip tapers to a fine diameter in intact vibrissae”, but no diameter values are reported. Yanli et al. [28] survey vibrissa shape in more than 20 mammalian species. They state that vibrissae are “conical” in all species examined, and for 23 species quote estimates for the basal diameter. They do not, however, provide a value for the tip diameter. To our knowledge, quantitative measurements are only available for rats and mice, where the ratio of vibrissae base to tip diameter is ∼15 [24], [29].

Measurements of whisker taper are complicated by the fact that whisker tips frequently erode, bend, fray, or break off during normal use. Published evidence on this is scarce, but there are several relevant papers. First, Ibrahim & Wright [29] measured weekly whisker growth in mice and rats and observed several breaks, though breakage rates were not quantified. Second, Sokolov & Kulikov [2] made a microscopic examination of the whiskers of nine rodent species and noted that the tips of “nearly all” vibrissae showed signs of wear. Third, Greaves et al. [30] studied the growth of whiskers in captive gray seals (*Halichoerus grypus*) by measuring the length of whiskers on a biweekly basis. Over the six month study period, about 35% of all whisker measurements gave a value lower than the value recorded two weeks previous, observations the authors interpreted as whisker breaks. Fourth, Neimark et al. [25] measured the dimensions of 18 mystacial (upper lip) vibrissae from the left side of a rat. Their whiskers (see their Table 1) can be placed into two classes: those with a tip diameter of 6 µm or less (10 out of 18, 56%) and those with a tip diameter of 12 µm or more (8 out of 18, 44%). Considering that other groups report a tip diameter of ∼5 µm for rat whiskers [24], [29], we suggest that whisker tips in the larger class represent breaks (the tapered shape means that a broken tip will be wider). The main consequence for the discussion here is that the normal use of whiskers involves breaks and erosion that may dramatically increase the tip diameter.

**Table 1:** Whisker taper.

Species	Common name	Taper (*R*_B_/*R*_T_)
Felis catus	domestic cat	7,10,13
Halichoerus grypus	gray seal	12,12,14
Martes pennanti	fisher	10,11,14
Mus musculus, var C3H	mouse	14,16,20,22,24
Panthera tigris	tiger	4,10,20
Procyon lotor	racoon	6,7,9
Puma concolor	cougar	4,8,11
Rattus norvegicus, var Wistar	rat	10,14,15,21,22
Taxidea taxus	badger	4,7,7
Ursus americanus	black bear	7,7,8
Vulpes vulpes	red fox	14,22,30

Measured taper (base radius/tip radius) for cheek vibrissae on preserved pelts. Typically, three of the longest vibrissae were selected. Data for rat and mouse are estimated from Ref. [29].

The fact that whisker breaks are common raises an interesting question about the possible selective advantage of tapered whiskers. Breaks occur near the tip, where the whisker is relatively narrow. A whisker of uniform diameter having the same mass would be much thicker at the tip, and would presumably break less often. One might therefore ask whether whisker taper provides some compensatory selective advantage that has led to its preservation during mammalian evolution. We will consider this possibility in the Discussion section.

In subsequent sections, we analyze the mechanics of isolated whiskers, and suggest that the taper may indeed provide some advantages for whiskers in their role as transducers. Throughout, we will compare the mechanical behavior of a tapered whisker model with an analogous untapered (cylindrical) whisker.

## Results

### Whisker Taper

To determine typical values for whisker taper, we measured whiskers attached to preserved pelts in the mammal collection at the University of Massachusetts, Amherst. Results are shown in Table 1. It is clear that whiskers are tapered across diverse genera, with a base to tip diameter ratio of ∼10. The occasional values well below 10 probably indicate whiskers with broken tips, although in the case of preserved pelts we cannot determine whether the breaks occurred post mortem.

**Figure 1:**
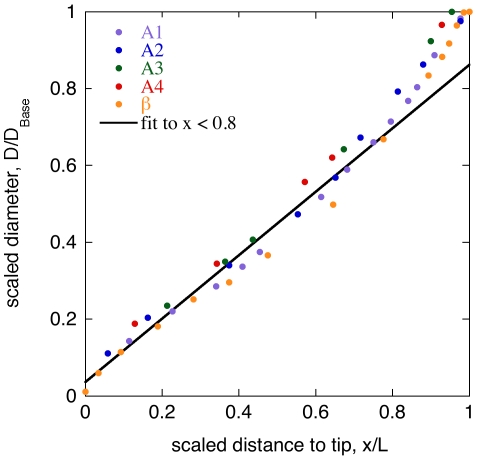
Whisker taper. Profiles of five whiskers from a Wistar rat (data from [29]), with *x* and *y* scaled by total whisker length and basal diameter respectively. Labels A1 through A4 and β indicate whisker locations on the mystacial pad. Line is the best fit to all points with scaled length less than 0.8, fit equation, *y* = 0.036+0.825 *x*.

We also wanted to characterize the linearity of the taper. Fig. 1 replots data reported by Ibrahim and Wright [29] for the first whisker row of a Wistar rat. The length and diameter have been scaled to provide a “typical” whisker profile. We see that the whisker profile is approximately linear, excepting some swelling close to the follicle. This is in agreement with [24] and [31], who approximate a rat whisker as a truncated cone (that is, a cone with the tip cut off). In this model, whisker profile is characterized by three numbers: length, base diameter, and tip diameter. The main variable of interest here is the degree of taper, which we define as the ratio of base to tip diameters.

### Maximum Deflection and Protraction

As we noted in the introduction, many mammal species use their whiskers to actively palpate an object, while in other species the whisker has a fixed direction and functions as a passive sensor. We will consider both situations, but begin with the passive case.

**Figure 2:**
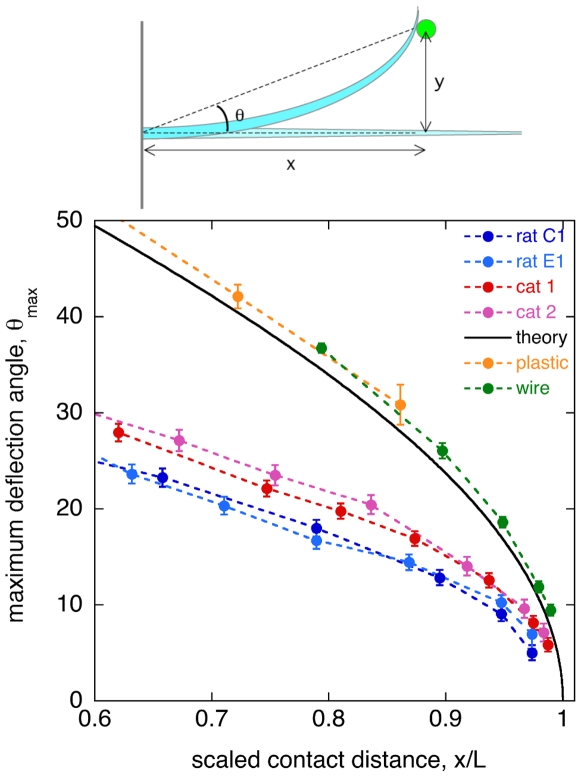
Maximal whisker deflection. Top: sketch of a passive whisker (blue) deflected by contact with an object (green). The deflection angle *θ* is the angle between the direction of the whisker base and the direction of the object. Bottom: maximum deflection angle as a function of normalized object distance *x/L* for two cat whiskers, two rat whiskers, and two artificial whiskers cut from steel wire or plastic fishing line. Solid line is the prediction of elastic beam theory for an untapered whisker under large deflections [47], assuming a frictionless contact between whisker and object. Error bars show standard errors (n = 4), and in some cases are smaller than the symbols.

Consider an isolated, straight, passive whisker as a probe for the direction of an object. Because the whisker is flexible, there will typically be a deflection angle *θ* between the basal direction of the whisker and the direction of the object in contact (Fig. 2). As described in the Methods section, we measure the maximal range of deflection angles that a whisker can sustain. Figure 2 shows typical results for the mystacial whiskers of rats and domestic cats, and for untapered model whiskers cut from steel wire or plastic fishing line. We find that, for a given object distance, the maximum deflection angle of a tapered whisker is smaller by about 50% as compared with an untapered whisker.

The theory of elastic beams predicts that a plot of maximal deflection angle, scaled as shown in Fig. 2, should be independent of the whisker length, diameter, and Young's modulus, and depend only on the whisker taper and the amount of friction at the contact point (see File S1). Our experiments are in approximate agreement with this independence. First, maximal deflections for the tapered whiskers of cat and rat differ from each other by less than ∼10°, and are clearly distinguished from the untapered model whiskers. Second, the maximal deflections of wire and fishing line compare well with each other and with the theoretical curve for a frictionless, cylindrical beam (the experimental values are somewhat larger than the theoretical curve, presumably due to friction between the pin and the whisker).

The small deflection angles observed for tapered whiskers might seem counterintuitive. Because the bending stiffness of a cylindrical beam increases with the fourth power of the diameter, tapered beams will be more flexible close to the tip, and thus more easily deflected. However, this does not result in a greater overall deflection. Instead, it tends to concentrate the curvature of the whisker into a zone near the tip, so that the whisker “flicks” past an obstacle without requiring globally high curvature.

**Figure 3:**
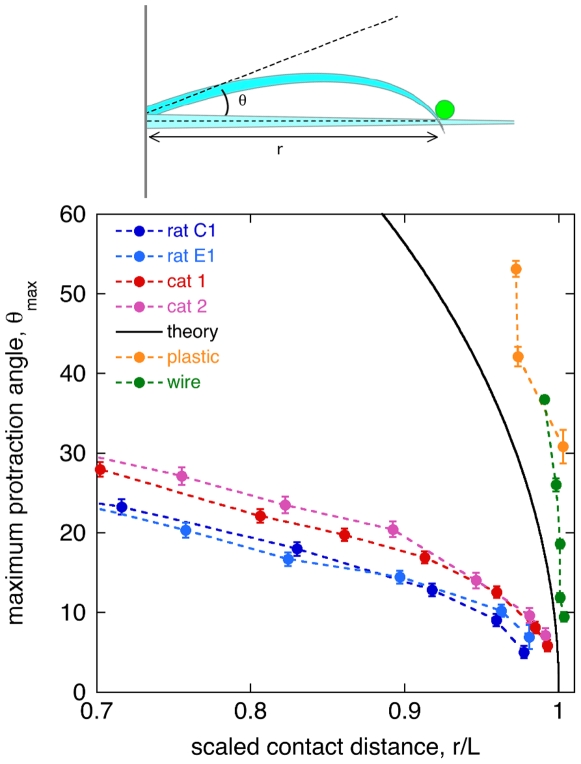
Maximal whisker protraction. Top: sketch of an active whisker (blue) undergoing protraction at its base during sustained contact with a stationary object (green). The protraction angle *θ* is defined relative to the surface normal. Bottom: re-display of the data shown in Fig. 1, to show the maximum protraction angle as a function of normalized object distance *r/L*. Symbols as in Fig. 2.

Now we consider active palpation of an object (Fig. 3). In this case the whisker comes into contact with a stationary object, and the whisker base is protracted by the muscles of the follicle, causing the whisker to bend. In Fig. 3, we re-plot the data gathered in the deflection experiment to show the maximum angle of protraction achieved before the whisker tip flicks past the object in contact. We see a large difference between the tapered whiskers and untapered model whiskers. Consider the example of a whisker that encounters an object at a normalized distance of 0.8. A tapered whisker would flick past the object with a protraction angle of ∼20°, while an untapered whisker would require a protraction angle larger than 60°.

### Rotational Stiffness

As we describe in the introduction, Szwed et al. [14] apply a contact to whiskers at 30%, 60%, or 90% of the distance from the whisker base, and observe two kinds of neuron encoding. As the contact is moved closer to the face, some cells increase their spike rate, and a higher proportion of touch-activated cells become active. Although the means by which distance discrimination is accomplished is not clear, the best available theory is based on the rotational stiffness. This quantifies the magnitude of the force generated by the FSC to protract the whisker base through, say, one additional degree of angle, *after* the whisker has made contact with an object. Closer contacts impose a higher rotational stiffness.

The rotational stiffness is defined as the derivative with respect to angle of the bending moment at the follicle, *K* = *dM*/*dθ*. For simplicity, Kaneko [22] and Solomon et al. [23] work with expressions valid for small protraction angles (*θ*<∼14°), an approximation that appears to be valid for rat whisking behavior [4], [31]. Solomon et al. [23] note that animal nervous systems are better at detecting rates of change rather than absolute values, and so suggest that the relevant biological variables for distance determination are the rate of change of the moment and the rate of change of the protraction angle (see their online Supplemental file). However, they show that the rotational stiffness *K* characterizes the relationship between these two rates.

**Figure 4:**
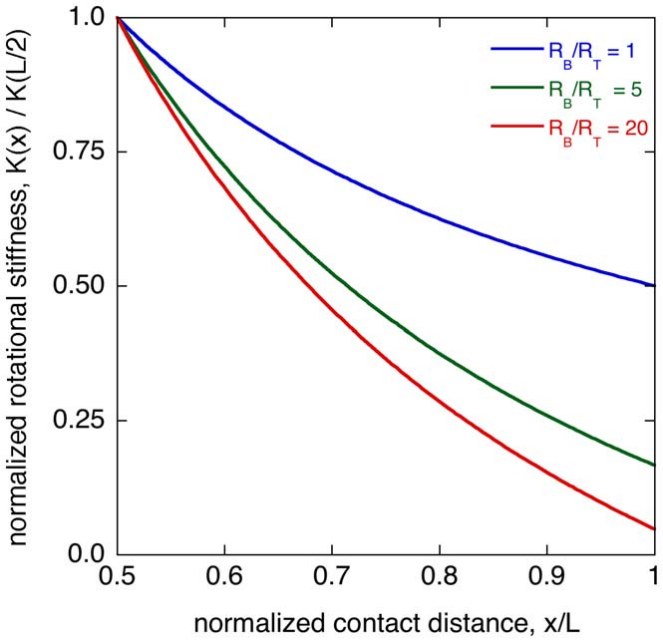
Rotational stiffness. Normalized rotational stiffness, K(x)/K(L/2), calculated for various truncated conical elastic beams. Top to bottom, the three curves correspond to tapers (*R*_B_/*R*_T_) of 1 (the untapered case), 5, and 20. We only show the distal half of the whisker, since whisker contacts are distal during natural behaviors [5].

The rotational stiffness of a truncated conical whisker in contact with an object at distance *x* from the whisker base is 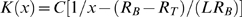, where *L* is the whisker length, *R_T_* and *R_B_* are the radii at the whisker tip and base respectively, and the constant 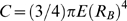, where *E* is the Young's modulus [23]. For an untapered beam, the equivalent expression is 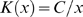 [22]. Closer objects produce larger and more rapid changes in the moment, so *K* increases with decreasing *x*. Figure 4 shows the rotational stiffness calculated for tapered and untapered whiskers. Between the whisker midpoint and the tip, the rotational stiffness of an untapered whisker varies by only a factor of 2, but for tapered whiskers the ratio is much larger: 6 and 21 for base to tip ratios of 5 and 20 respectively.

### Robustness of the Resonant Frequency

As discussed in the introduction, the whisker shaft functions as a mechanical oscillator during texture discriminations, with a resonant frequency that may be understood using the classical theory of elastic beams [24], [25]. Andermann et al. [15] identified neurons in the Vg and S1 that respond to vibrations within ∼40 HZ of the resonant frequency, and that show no response (above background) outside this range. They concluded that this frequency tuning was achieved via whisker resonance. The amplitude and velocity of whisker vibration is enhanced near the resonant frequency, and the mechanoreceptors of the follicle respond to this increased amplitude and/or velocity. This suggests that, if physical damage to the whisker tip leads to a change in resonant frequency, the neural circuits of Vg and S1 will respond to the wrong frequency components in whisker stimuli.

**Figure 5:**
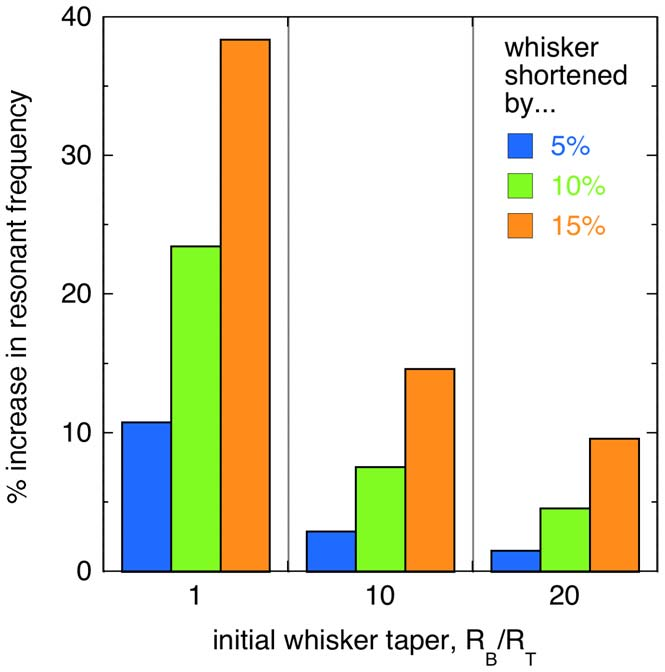
Resonant frequency robustness . Graph showing the percentage increase in the resonant frequency of a model whisker after a break that decreases the length by 5, 10, or 15%. Values are calculated using Eqns. (1) and (2), for an untapered elastic beam (*R*_B_/*R*_T_ = 1) or a tapered elastic beam (*R*_B_/*R*_T_ = 10 and 20).

The resonant frequency of a tapered whisker is relatively robust under tip breaks, compared to the resonant frequency of an untapered model whisker. As discussed in the Methods section, the theory of elastic beams can be used to express the fundamental resonant frequency of a cylindrical or tapered beam in terms of its density, Young's modulus, base and tip radii, and length [24], [32]. We use this expression to calculate the change in the resonant frequency of a whisker if the tip breaks (Fig. 5). Consider, for example, a typical whisker with a base to tip diameter ratio of 10. If the distal 10% of the whisker breaks off, beam theory predicts an increase in the resonant frequency of 8%. An untapered whisker suffering the same break will increase in frequency by 23%. Conversely, a whisker with a base to tip diameter ratio of 20 shows a smaller change, with a resonant frequency increase of just 5%. In general, the resonant frequency of a tapered whisker is less sensitive to breaks than an untapered whisker, and sharply tapered whiskers are less sensitive than blunt tapered whiskers.

## Discussion

Whiskers are absent in monotremes, so it is believed that whiskers evolved in the therian mammals about 120 million years ago [21]. We know of no fossilized whiskers, so it is unclear whether the whiskers of early therians were tapered. However, published data on extant mammals [1], [28], [29], and our own survey (Table 1), suggests that the tapered profile is widespread. As far as we know, the only exception to the typical “truncated cone” shape occurs in the superfamily Pinnipedia (seals and walrus). These have whiskers that are oval in cross section, as compared to the circular cross section of terrestrial mammals [33]. More dramatic is the shape of whiskers in phocid seals, a subgroup of the pinnipeds. Their whiskers do taper from base to tip, but with periodic oscillations in diameter that give the whisker a wavy or “beaded” profile [33], [34]. It seems likely to us that differences in the whisker shape of terrestrial mammals and pinnipeds reflect the differences in terrestrial and marine environments. For example, phocid seals can use their whiskers to detect small water currents produced by prey animals [3]. No equivalent behavior has yet been shown in a terrestrial mammal, possibly because the much lower viscosity and density of air prevents this mode of whisker use. In the discussion that follows, we focus on terrestrial mammals.

Exceptions to the conical shape of a whisker are important for another aspect of our discussion. Because we are arguing that tapered whiskers may have some selective advantages, it is important to emphasize that the taper does not reflect fundamental constraints on the development or physiology of the follicle. For example, the oval cross section of pinnipeds and the beaded profile of phocid seals demonstrate that evolution can select for different whisker shapes. Looking beyond vibrissae, the surprising variety of cross-sections in the non-vibrissal hair of mammals [6], the fact that pelage (body) hair is untapered except near the tip [35], and even the evolution of feathers [36], show that integumentary appendages grown from follicles can exhibit a wide range of morphologies. Thus, we suggest that the widespread occurrence of tapered whiskers does not reflect a fundamental constraint on follicle development or physiology.

In the paragraphs that follow, we review our analysis of whisker mechanics and suggest ways that the taper may provide a selective advantage. Before pursuing this, it is helpful to mention two aspects of whisker mechanics and physiology that we will neglect below. First, we neglect whisker curvature and consider only straight whiskers. A discussion of whisker curvature may be found in Birdwell et al. [31], who consider the effect of curvature on whisker mechanics and argue that the effects are generally small. Second, we neglect the possibly important role of follicle compliance. The deflection and vibration properties of the whisker depend on, and may be modulated by, the mechanical properties of the FSC [24], [25].

Now we consider each subsection of the Results in turn. The following discussion is necessarily speculative, since we are trying to apply observations on isolated and model whiskers to a consideration of animal behavior and evolutionary advantage. We will distinguish between passive vibrissae, whose angle of emergence from the skin is fixed, and active vibrissae, whose angle is under voluntary control. Most published work on vibrissae has focused on the active mystacial vibrissae of rats, but it should be noted that passive whiskers are common in herbivores, and that species with active whiskers typically have additional, passive whiskers at locations other than the mystacial pad [2], [6], [37].

### Whisker Taper

We suggest that one of the main advantages of whisker taper, at least for active whiskers, is to provide a small diameter at the whisker tip, to allow for a finer probe of small surface features. Recent studies of texture discrimination in rats highlight the importance of stick-slip events: as the whisker is swept over a surface, the tip of the whisker is briefly in static contact with the surface, and subsequently slips before a new static contact is established [17], [18], [19], [26]. Static contacts are due to a combination of mechanical pinning and static friction, and a smaller whisker tip diameter will presumably allow the whisker to make static contacts with smaller surface features.

A comparison of whisker tip diameter with measurements of whisker tactile acuity is consistent with this view. Carvell et al. [9] found that rats could reliably distinguish a smooth surface from one with 30-µm deep grooves. Arabzadeh et al. [18] measured whisker vibration and neuron firing patterns during surface sweeps, and found distinct responses for sandpapers with a mean grain size of 15 and 35 µm (Jadhav et al. [17] subsequently returned to this protocol and identified stick-slip events as key stimuli). Thus, surface features in the range of 15 to 30 µm can influence rat whisker kinetics and performance on sensory tasks. This is to be compared with a typical unbroken tip diameter of ∼5 µm [24], [29] and an observed range of broken tip diameters 12 to 35 µm [25]. Whisker tip diameters are thus somewhat smaller or comparable to the known limits of surface feature discrimination in rats.

If the smallness of the tip diameter is important for proper whisker function, this may help explain why whiskers are routinely shed and replaced. The growth and replacement cycle lasts about 5 weeks in mice, and 8 weeks in rats [29] (the whiskers are not shed synchronously with each other or with a molt of pelage hair). Sokolov & Kulikov [2] previously suggested that the whisker replacement cycle was an adaptation to limit the deleterious effects of wear at the tip, although they did not speculate about how wear might limit whisker performance. We suggest that whisker tip erosion or breaks may decrease the effectiveness of whiskers during fine texture discriminations by gradually increasing the tip diameter.

Of course, taper is a statement about the relative diameter of whisker tip and base. The preceding argument may help explain why whisker tips are narrow, but not why the whisker base should be relatively broad. Recall that the bending modulus of an elastic beam increases with the fourth power of diameter. It is thus possible that the larger basal diameter is necessary to maintain the overall rigidity of the whisker, so that the muscles of the follicle can accurately control the location of the tip.

### Maximum Deflection and Protraction

In the case of passive whiskers, we consider the case of an object moving relative to the whisker, and measure the maximum deflection angle between the direction of the whisker base and the direction of the object in contact (Fig. 2). The maximum deflection angle for a tapered whisker is substantially smaller than that for an untapered model whisker. As a result, the volume of space accessible to a passive tapered whisker is only about 1/4th that of an untapered whisker. In this sense, tapered whiskers have a higher spatial acuity.

The role of passive whiskers in behavior, and their neural correlates, have not been studied as thoroughly as active whiskers. However, likely roles for passive whiskers are (1) to help the animal locate objects near the body and orient in response, and (2) to function as “guard” vibrissae in various avoidance responses, to protect the face and body against abrasion or attack [1], [2]. In both cases, we suggest that the higher spatial acuity of tapered whiskers may provide a selective advantage. In particular, the speed and accuracy of avoidance behaviors triggered by guard vibrissae may benefit from this acuity. More research on passive whiskers will be required before firmer conclusions can be drawn.

For the case of active sensing (Fig. 3), we find that tapered whiskers can sustain object contact over a much narrower range of protraction angles than untapered model whiskers. This initially suggested to us that tapered whiskers would provide an improved spatial acuity during palpations. However, the maximal protraction angle before flick past may not be a relevant criterion for this task. Whisker protraction during object palpation appears to be limited to angles less than ∼5°, smaller than our measured flick past threshold in rats except very near the tip [4], [7]. On the other hand, stick-slip events observed during texture discriminations may provide a better application of the flick past geometry considered here [17], [18], [19], [26] (see especially the whisker tracking images of Ritt et al. [26]). By limiting the size of whisker deflections during a stick event, taper may help the follicle control the movement of the whisker tip across a surface.

### Rotational Stiffness

As described above, several groups [22], [23], [31] have suggested that rotational stiffness *K* is a plausible proxy for the distance *d* between the whisker base and the point of object contact. We noted that an untapered whisker has a relatively weak dependence of *K* on *d*, varying by a factor of 2 between the whisker midpoint and the tip, as compared with tapered whiskers, which vary by much larger amounts (Fig. 4). In an analysis of robotic whiskers, Kaneko [22] noted that the interpretation of rotational stiffness in real-world settings would be complicated by object compliance (softness), friction, and curvature. For example, a low value for *K* could reflect contact with a nearby, compliant object, or a more distant, harder object. To resolve this difficulty, Kaneko (who considers only untapered model whiskers) proposed a revised distance proxy that requires more than one contact event with the object. While rat whisking behavior does provide stereotyped and repeated contact with an object, this is not the case with non-whisking mammals. In addition, the observation of neuron populations in rats that respond to object distance during a single, sustained contact [14] suggest that repeated contacts are not required. We suggest instead that the steeper dependence of *K* on *d* for tapered whiskers might be a simple way to minimize the ambiguity introduced by object compliance, curvature, and friction.

One caveat to this proposal is the possible importance of a detection threshold for mechanoreceptors in the follicle. Because of the steepness of the *K* -d curve for tapered whiskers (Fig. 4), tissue stresses in the follicle due to object contact near the whisker tip will be relatively small. Sub-threshold stresses would limit the advantage to be gained from a tapered whisker. Further progress will require a quantitative analysis of the tissue stresses, perhaps using a finite element model similar to Ref. [38] for fingertips.

### Robustness of the Resonant Frequency

In the section on whisker resonant frequencies, we noted that the principle resonant frequency of a tapered whisker is relatively robust under whisker breaks. That is, the resonant frequency changes by a small percentage as compared with similar breaks in an untapered whisker (Fig. 5). At first, this might appear to provide a substantial advantage during texture discrimination tasks. As described by Neimark et al. [25], a whisker array swept across an object may provide texture information as a Fourier transform, with different whiskers oscillating at different frequencies. Thus, the robustness of each whisker under tip breaks would be a way to preserve the sensory encoding scheme of the array. However, an untapered whisker with the same mass will be thicker at the tip, and so presumably less likely to break at all. In the absence of additional information, we can only suggest that whisker taper is selectively neutral as regards resonant mechanical oscillations during texture sensing.

The idea that integumentary appendages adjacent to mechanoreceptors have been shaped by evolution to improve their tactile sensitivity or performance is not new. For example, it has been independently proposed twice that the epidermal ridges of human fingertips, and the underlying arrangement of papilla, serve to focus tissue stresses at the sites of mechanoreceptors [38], [39]. The tapered shape of the lobster antenna was recently analyzed from this perspective as well [40] (note that the lobster antenna is innervated, and so not an analog for the whiskers considered here).

In this paper we have analyzed the mechanical properties of isolated whiskers and discussed these properties as they might relate to various proposals for whisker sensing *in vivo*. By contrasting the properties of a tapered with an untapered model whisker, we have argued that the taper itself may be functionally important. In the absence of additional experiments on live animals, these proposals remain speculative. However, we suggest that the widespread and possibly universal occurrence of whisker taper in terrestrial mammals reflects the preservation of whisker taper during therian evolution, due to the selective advantages of the tapered profile. It may also explain the parallel evolution of tapered touch-sensitive bristles and hairs in arthropods (e.g., the macrochaetes and microchaetes of *Drosophila*) [41], [42], [43]. Lastly, these results suggests that robotic whisker systems designed to operate under diverse environmental conditions would benefit from the incorporation of tapered whiskers [22], [23], [44], [45].

## Methods

### Whisker Taper

Whiskers in Table 1 were measured on preserved pelts from the mammal collection at the University of Massachusetts, Amherst. Using digital calipers we measured several dimensions of whiskers while still attached to the pelts. Three of the longest attached whiskers were measured for each pelt. Dimensions included length (without correction for curvature), basal diameter (as near to the skin as possible), and tip diameter. Since the calipers had a resolution of 10 µm, and since the whisker was tapered, we regard tip diameter values only as plausible upper bounds on the true value.

### Whisker Deflection Theory

The theoretical maximum deflection angle for a frictionless, untapered beam was found using a shooting method algorithm [46] to solve the equations of an elastic beam under large deflections [31], [47], [48]. Further details of this calculation may be found in File S1.

### Whisker Deflection Experiment

Rat whiskers were provided by B. Quist and M. Hartmann. Following the procedure of [31], whiskers were plucked from a female Sprague-Dawley rat, mass 274 g, under full anesthesia for an unrelated experiment. Cat whiskers were not plucked. Instead, shed whiskers were collected and donated by the owners of several domestic cats. The labels cat 1 and cat 2 (used in Figs. 2 and 3) refer respectively to whiskers from a 4 y.o. male and a 13 y.o. female, both 7 kg. Cat whiskers were only used if they were long and straight enough for us to conclude that they were mystacial (upper lip) whiskers. Model untapered whiskers were cut from lengths of steel wire and plastic fishing line.

The maximum deflections of whiskers were measured via the following method. The whisker is clamped at its base and a metal pin is moved perpendicular to the whisker at a fixed horizontal distance *x* from the clamp (Fig. 2). As the vertical distance *y* increases, the whisker or wire eventually slips past the pin and returns to its equilibrium shape. The maximum deflection angle is 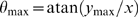. Whiskers with detectable curvature were clamped so that the curvature was perpendicular to the *x-y* plane. We confirmed that this produced symmetric plots of *θ*_max_. To re-express the data as a protraction angle (Fig. 3), we plotted *θ*_max_ versus the object distance, 
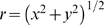
.

### Resonant Frequency

The resonant frequencies of a cylindrical beam and a truncated cone are classic problems in elasticity theory [32]. The exact numerical result depends on the boundary conditions, but following previous reports we focus on the case of a beam clamped at the wide end and free to vibrate at the tapered end [24], [25]. Other boundary conditions, or a consideration of higher order resonances, will change the numbers somewhat but will not change the qualitative conclusions in the Discussion section. The fundamental resonant frequency *f* of a cylindrical or tapered beam of length *L*, base radius *R_B_*, uniform density *ρ*, and Young's modulus *E* is
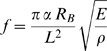
(1)where *α* is a dimensionless number that depends on the ratio of the tip and base diameters, *R_T_* and *R_B_* [24], [32]. For a cylindrical beam, *α* = 3.516 and for a conical beam with no truncation, *α* = 8.718. Intermediate cases fall between these values. Following Conway et al. [32], we used Maple (v.10, Waterloo Maple, Canada) to solve for the value of *α* at a range of taper values, and then used Kaleidagraph (v.3.6.4, Synergy Software) to fit a polynomial for large taper. The expression
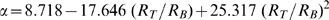
(2)is accurate to 1% for *R_T_*/*R_B_*<0.25.

## Supporting Information

File S1 Detailed calculation of the deflection of an elastic beam.
(0.26 MB PDF)

## References

[1] Ahl AS (1986). The role of vibrissae in behavior: a status review.. Veterinary Research Communications.

[2] Sokolov VE, Kulikov VF (1987). The structure and function of the vibrissal apparatus in some rodents.. Mammalia.

[3] Dehnhardt G, Mauck B, Bleckmann H (1998). Seal whiskers detect water movements.. Nature.

[4] Carvell GE, Simons DJ (1995). Task and subject related differences in sensorimotor behavior during active touch.. Somatosensory & Motor Research.

[5] Mitchinson B, Martin CJ, Grant RA, Prescott TJ (2007). Feedback control in active sensing: rat exploratory whisking is modulated by environmental contact.. Proceedings of the Royal Society of London B Biological Sciences.

[6] Chernova OF (2006). Evolutionary aspects of hair polymorphism.. Biology Bulletin.

[7] Krupa DJ, Matell MS, Brisben AJ, Oliveira LM, Nicolelis MAL (2001). Behavioral Properties of the Trigeminal Somatosensory System in Rats Performing Whisker-Dependent Tactile Discriminations.. Journal of Neuroscience.

[8] Knutsen PM, Pietr M, Ahissar E (2006). Haptic Object Localization in the Vibrissal System: Behavior and Performance.. Journal of Neuroscience.

[9] Carvell GE, Simons DJ (1990). Biometric Analyses of Vibrissal Tactile Discrimination in the Rat.. Journal of Neuroscience.

[10] Hipp J, Arabzadeh E, Zorzin E, Conradt J, Kayser C (2006). Texture Signals in Whisker Vibrations.. Journal of Neurophysiology.

[11] Shoykhet M, Doherty D, Simons DJ (2000). Coding of deflection velocity and amplitude by whisker primary afferent neurons: implications for higher level processing.. Somatosensory & Motor Research.

[12] Szwed M, Bagdasarian K, Ahissar E (2003). Encoding of vibrissal active touch.. Neuron.

[13] Andermann ML, Moore CI (2006). A somatotopic map of vibrissa motion direction within a barrel column.. Nature Neuroscience.

[14] Szwed M, Bagdasarian K, Blumenfeld B, Barak O, Derdikman D (2006). Responses of trigeminal ganglion neurons to the radial distance of contact during active vibrissal touch.. Journal of Neurophysiology.

[15] Andermann ML, Ritt J, Neimark MA, Moore CI (2004). Neural Correlates of Vibrissa Resonance: Band-Pass and Somatotopic Representation of High-Frequency Stimuli.. Neuron.

[16] Andermann ML, Moore CI (2008). Mechanical resonance enhances the sensitivity of the vibrissa sensory system to near-threshold stimuli.. Brain Research.

[17] Jadhav SP, Wolfe J, Feldman DE (2009). Sparse temporal coding of elementary tactile features during active whisker sensation.. Nature Neuroscience.

[18] Arabzadeh E, Zorzin E, Diamond ME (2005). Neuronal Encoding of Texture in the Whisker Sensory Pathway.. PLoS Biology.

[19] von Heimendahl M, Itskov PM, Arabzadeh E, Diamond ME (2007). Neuronal Activity in Rat Barrel Cortex Underlying Texture Discrimination.. PLoS Biology.

[20] Gottschaldt K-M, Iggo A, Young DW (1973). Functional characteristics of mechanoreceptors in sinus hair follicles of the cat.. Journal of Physiology.

[21] Brecht M, Preilowski B, Merzenich MM (1997). Functional architecture of the mystacial vibrissae.. Behavioural Brain Research.

[22] Kaneko M, Kanayama N, Tsuji T (1998). Active antenna for contact sensing.. IEEE Transactions on Robotics.

[23] Solomon JH, Hartmann MJ (2006). Robotic whiskers used to sense features.. Nature.

[24] Hartmann MJ, Johnson NJ, Towal RB, Assad C (2003). Mechanical characteristics of rat vibrissae : resonant frequencies and damping in isolated whiskers and in the awake behaving animal.. Journal of Neuroscience.

[25] Neimark MA, Andermann ML, Hopfield JJ, Moore CI (2003). Vibrissa resonance as a transduction mechanism for tactile encoding.. Journal of Neuroscience.

[26] Ritt JT, Andermann ML, Moore CI (2008). Embodied Information Processing: Vibrissa Mechanics and Texture Features Shape Micromotions in Actively Sensing Rats.. Neuron.

[27] Ahl AS (1987). Relationship of vibrissal length and habits in the Sciuridae.. Journal of Mammalogy.

[28] Yanli B, Wei Z, Yanchun X, Jun Z, Xiaoming T (1998). Relationship between structure and function of mammalian vibrissa.. Journal of Forestry Research.

[29] Ibrahim L, Wright EA (1975). The growth of rats and mice vibrissae under normal and some abnormal conditions.. Journal of Embryology and Experimental Morphology.

[30] Greaves DK, Hammill MO, Eddington JD, Pettipas D, Schreer JF (2004). Growth rate and shedding of vibrissae in the gray seal, *Halichoerus grypus*, a cautionary note for stable isotope diet analysis.. Marine Mammal Science.

[31] Birdwell JA, Solomon JH, Thajchayapong M, Taylor MA, Cheely M (2007). Biomechanical Models for Radial Distance Determination by the Rat Vibrissal System.. Journal of Neurophysiology.

[32] Conway HD, Becker ECH, Dubil JF (1964). Vibration Frequncies of Tapered Bars and Circular Plates.. Journal of Applied Mechanics.

[33] Perrin WF, Wursig B, Thewissen JGM (2008). Encyclopedia of Marine Mammals, 2nd edition.

[34] Dehnhardt G, Kaminski A (1995). Sensitivity of the mystacial vibrissae of harbor seals (Phoca vitulina) for size differences of actively touched objects.. Journal of Experimental Biology.

[35] Hausman LA (1920). Structural characteristics of the hair of mammals.. American Naturalist.

[36] Prum RO, Brush AG (2002). The evolutionary origin and diversification of feathers.. The Quarterly Review of Biology.

[37] Pocock RI (1914). On the facial vibrissae of mammalia.. Proceedings of the Zoological Society of London.

[38] Maeno T, Kobayashi K, Yamazaki N (1998). Relationship between the structure of human finger tissue and the location of tactile receptors.. JSME International Journal.

[39] Cauna N (1954). Nature and function of the papillary ridges of the digital skin.. Anatomical Record.

[40] Barnes TG, Truong TQ, Adams GG, McGruer NE (2001). Large Deflection Analysis of a Biomimetic Lobster Robot Antenna due to Contact and Flow.. Transactions of the ASME.

[41] McIver SB (1975). Structure of cuticular mechanoreceptors of arthropods.. Annual Review of Entomology.

[42] Tilney LG, DeRosier DJ (2005). How to make a curved Drosophila bristle using straight actin bundles.. Proceedings of the National Academy of Sciences.

[43] Kernan MJ (2007). Mechanotransduction and auditory transduction in Drosophila.. European Journal of Physiology.

[44] Kim D, Möller R (2007). Biomimetic whiskers for shape recognition.. Robotics and Autonomous Systems.

[45] Pearson MJ, Pipe AG, Melhuish C, Mitchinson B, Prescott TJ (2007). Whiskerbot: a robotic active touch system modeled on the rat whisker sensory system.. Adaptive Behavior.

[46] Press WH, Flannery BP, Teukolsky SA, Vetterling WT (1989). Numerical Recipes.

[47] Landau LD, Lifshitz EM (1986). Theory of Elasticity, 3rd ed.

[48] Barber DJ, Loudon R (1989). An introduction to the properties of condensed matter.

