# Supplementary material for: The Advantages of a Tapered Whisker
Source: PLoS One. 2010 Jan 20;5(1):e8806. doi: 10.1371/journal.pone.0008806 (PMC2808387; doi:10.1371/journal.pone.0008806)
Supplement: File S1 — Detailed calculation of the deflection of an elastic beam. (0.26 MB PDF) [file pone.0008806.s001.pdf]

The Advantages of a Tapered Whisker

C. M. Williams & E. M. Kramer

Supplemental File 1

Beam theory equations

We follow standard treatments of elastic beam theory [1-3]. Consider an elastic beam deflected by a frictionless pin (Fig. A1). The midline of the beam is parameterized by the arclength s, with $s = 0$ at the origin of the x-y plane. The beam is deflected by a frictionless pin that makes contact with the beam at $s = s_p$. The deflection of the beam is completely characterized by the function $\theta(s)$, to be determined.

Consider a finite segment of the beam, between the origin and s (Fig. A1). The equations describing force and rotational equilibrium on this segment are respectively

$$N_0 = N(s)\cos(\theta(s)) + F(s)\sin(\theta(s)) \quad (A1)$$

$$F_0 = F(s)\cos(\theta(s)) - N(s)\sin(\theta(s)) \quad (A2)$$

$$M(s) = M_0 - xF_0 - yN_0 \quad (A3)$$

where $N(s)$ is the force tangent to the beam at s, $F(s)$ is the shear force, $M(s)$ is the bending moment (calculated about the point s), and the subscript 0 indicates values at the origin. It is helpful to solve Eqns. (A1) and (A2) for N and F

$$N(s) = N_0\cos(\theta(s)) - F_0\sin(\theta(s)) \quad (A4)$$

$$F(s) = F_0\cos(\theta(s)) + N_0\sin(\theta(s)) \quad (A5)$$

The coordinates x and y can be expressed as integrals of $\theta(s)$ via

$$x(s) = \int_0^s ds' \cos(\theta(s'))$$
$$y(s) = \int_0^s ds' \sin(\theta(s')) \qquad \text{(A6)}$$

Assuming the pin is frictionless, it can apply a shear force at the contact point, but no tangential force or bending moment. Thus, at the pin (s=s_P), we have the boundary conditions

$$N_P = 0 = N_0 \cos(\theta_P) - F_0 \sin(\theta_P) \qquad \text{(A7)}$$

$$F_P = F_0 \cos(\theta_P) + N_0 \sin(\theta_P) \qquad \text{(A8)}$$

$$M_P = 0 = M_0 - x_P F_0 - y_P N_0 \qquad \text{(A9)}$$

The shear force applied by the pin, F_P is unknown, and is solved for as part of a nonlinear eigenvalue problem.

Lastly, we have the relationship between curvature and moment

$$\frac{d\theta}{ds} = \frac{M(s)}{EI} \qquad \text{(A10)}$$

where E is the Young's modulus and I is the area moment of inertia. For a beam with circular cross section of radius R, $I = \pi R^4/4$.

Rewriting Eqn. (A7) gives $N_0 = F_0 \tan(\theta_P)$. Then, using (A8) and (A9) to eliminate F_0 and M_0 in favor of F_P, and substituting into (A3) gives

$$M(s) = F_P \left\{ \left[x_P - x(s) \right] \cos(\theta_P) + \left[y_P - y(s) \right] \sin(\theta_P) \right\} \qquad \text{(A11)}$$

substituting into Eqn. (A10) gives, at last

$$\frac{d\theta}{ds} = \frac{4}{\pi} \frac{F_P}{ER^4} \left\{ \left[x_P - x(s) \right] \cos(\theta_P) + \left[y_P - y(s) \right] \sin(\theta_P) \right\} \qquad \text{(A12)}$$

Eqns. (A6) and (A12) constitute the set of equations to be solved numerically for θ(s), subject to the starting boundary condition $\theta(0)=0$. For an untapered beam, R is

independent of s. For a tapered beam, $R(s) = R_B(1 - \gamma(s/L))$, where $\gamma = 1 - (R_T/R_B)$, R_B is the radius at the base of the beam, R_T is the radius at the tip of the beam and, and L is the total length of the beam.

From the deflection experiments, we know that the untapered beam maintains $\theta < 90°$ as the pin is dragged past. Thus, for a given x value for the pin (see Fig. 2), the largest y value will occur when the far end of the beam is exactly at the pin (i.e. when $s_P = L$).

Eqns. (A6) and (A12) for a frictionless beam can be simplified by expressing them in terms of the dimensionless variables $u = x/L$, $v = y/L$ and $w = s/L$

$$u(w) = \int_0^w dw' \cos(\theta(w'))$$

$$v(w) = \int_0^w dw' \sin(\theta(w'))$$

$$\frac{d\theta}{dw} = \frac{A}{(1-\alpha w)^4}\left\{\left[u_P - u(w)\right]\cos(\theta_P) + \left[v_P - v(w)\right]\sin(\theta_P)\right\} \qquad \text{(A13)}$$

where $A = 4F_P L^2/(\pi E R_B^4)$ is the dimensionless shear force at the pin. For a given taper, characterized by α, this presents a nonlinear eigenvalue problem for the set of four parameters (A, u_P, v_P, θ_P).

We solve the set of Eqns. (A13) for the untapered case ($\gamma = 0$), using standard shooting methods [4]. For a given choice of A, there is a unique set (u_P, v_P, θ_P) that satisfies the boundary condition with the pin at the tip of the beam, $w_P = 1$. Having solved this problem for a range of A values, we express the maximum scaled deflection $v_{P,max}$ directly in terms of the scaled contact distance u_P. In Fig. 2 we plot the maximum

deflection angle θ_{max} = atan($v_{P,max}/u_P$) vs. u_P. In Fig. 3 we plot θ_{max} vs. the scaled distance to the pin, $[(u_P)^2+(v_{P,max})^2]^{1/2}$.

Other choices for γ, not solved in this paper, will give different solutions for the eigenvalue problem. Thus, the maximum deflection and protraction angles, plotted as a function of scaled contact distance, depend on the taper through γ, and on friction with the pin, but are otherwise independent of E, R_B, and L.

References

1. Landau, L.D., and Lifshitz, E.M. (1986). Theory of Elasticity, 3rd ed., (Boston: Butterworth Heinemann).
2. Barber, D.J., and Loudon, R. (1989). An introduction to the properties of condensed matter, (Cambridge: Cambridge University Press).
3. Birdwell, J.A., Solomon, J.H., Thajchayapong, M., Taylor, M.A., Cheely, M., et al. (2007). Biomechanical Models for Radial Distance Determination by the Rat Vibrissal System. Journal of Neurophysiology *98*, 2439-2455.
4. Press, W.H., Flannery, B.P., Teukolsky, S.A., and Vetterling, W.T. (1989). Numerical Recipes, (New York: Cambridge University Press).

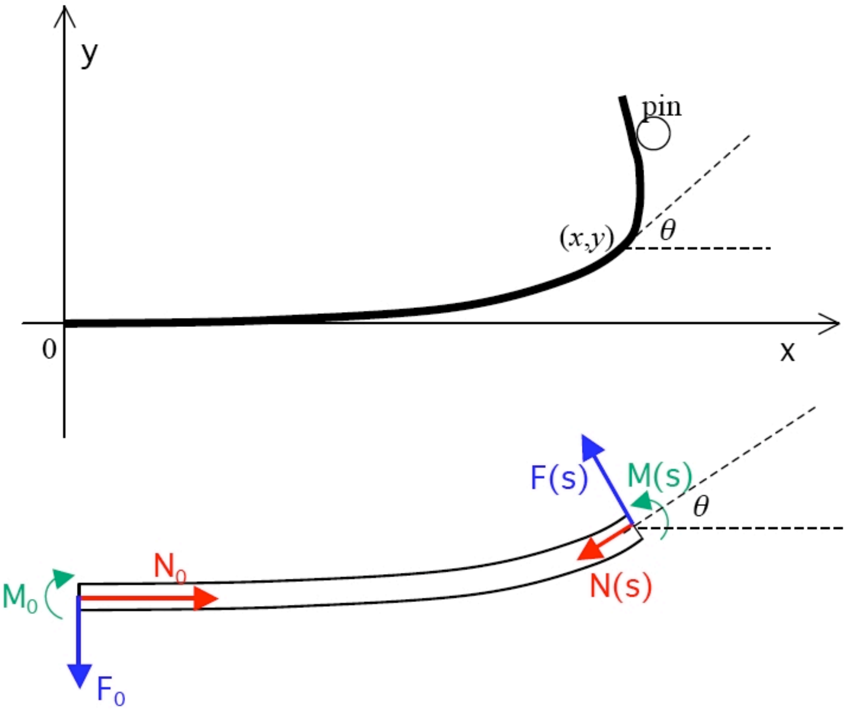

Figure A1. Elastic beam. Top: coordinates used in the calculation. θ is the angle between the beam tangent vector and the x-axis. Bottom: A segment of the beam, between the origin and s, showing the forces and moments applied to the ends. Red: normal forces. Blue: shear forces. Green: bending moments. Note that the bending moment at s is defined to be positive if it produces an upward curvature.
